# Fabrication of white light emitting diodes via high yield surface passivated carbon quantum dots doped with terbium

**DOI:** 10.1039/d2ra07890b

**Published:** 2023-01-11

**Authors:** Ravi Pratap, Vipul Vishal, Shilpi Chaudhary, Avanish Singh Parmar

**Affiliations:** a Department of Physics, Indian Institute of Technology (BHU) Varanasi Varanasi India asparmar.phy@iitbhu.ac.in; b School of Medical Science and Technology, Indian Institute of Technology Kharagpur Kharagpur India; c Department of Applied Sciences, Punjab Engineering College (Deemed to be University) Chandigarh India

## Abstract

Exploiting the unique characteristics of various materials to create novel hybrid materials opens up innovative possibilities for cutting-edge applications across numerous fields. Here, we have synthesized novel surface functionalized photoluminescent carbon quantum dots (CQDs) doped with a rare-earth element (Tb^3+^) for white light emitting diodes. High quantum yield CQDs were produced utilizing *Plumeria* leaves as a precursor using a one-step hydrothermal approach, and further, its optical characterization was thoroughly investigated. Herein, the functionalized CQDs demonstrate excitation-independent electroluminescence performance. The UV-LED chip and functionalized CQD were combined to create a device that emits cold white light with Commission Internationale de L'Eclairage coordinates of (0.33, 0.34), a corresponding correlated color temperature of 4995 K and color rendering index of 84.2.

## Abstract 2

Exploiting the unique characteristics of various materials to create novel hybrid materials opens up innovative possibilities for cutting-edge applications across numerous fields.

## Graphical abstract

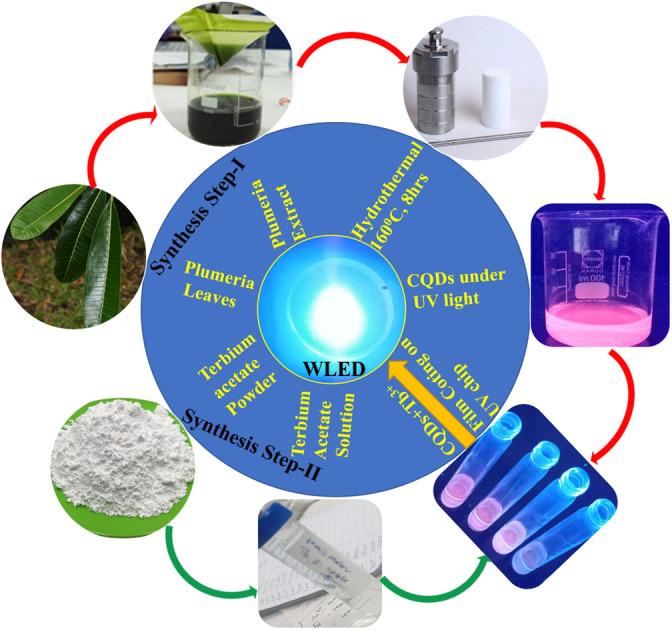

## 1. Introduction

Traditional light sources are being replaced by light emitting diodes (LEDs) owing to their long lifetime, high durability, compact size, good quantum yield, and efficient energy saving.^1–4^ In particular, white LEDs (WLEDs) are continuously researched since they have applications in lighting and liquid crystal display technology.^1,2^ At present, most commercial WLEDs are fabricated by phosphor (yellow/red) materials coated over a blue LEDs chip. The purpose of phosphor materials is to act as colour conversion layer. For the production of WLEDs, researchers recently fabricated a variety of phosphors.^5^ In order to create WLEDs, Jang *et al.*^6^ coated high fluorescent yellow phosphors materials like Y_3_Al_5_O_12_ : Ce^3+^ (YAG : Ce), Tb_3_Al_5_O_12_ : Ce^3+^ (TAG : Ce), and Sr_3_SiO_5_ : Eu^2+^ materials (SS : Eu) on the blue LED chip of InGaN. In an another study, Chen *et al.*^7^ reported that the Eu-activated LaSiO_2_N yellow phosphor can be used in the application of WLEDs. However, the key issue is the high reaction temperature and cost of the raw ingredients needed to synthesize phosphor compounds. As a yellow phosphor for WLED applications, several researchers have used heavy metal-containing semiconductor quantum dots (S-QDs) such as CdSe-QDs,^8^ CdSe/ZnS-QDs,^9^ and CdSe/ZnS/CdSe CdS/ZnS-QDs.^10^ These semiconductor QDs are not only highly toxic in nature but also require high energy consumption during the synthesis process. Therefore, the difficulties in manufacturing WLEDs remain a significant barrier. Li *et al.* used CdTe quantum dots and carbon dots (CDs) to create a warm white LED with such a CIE of (0.38, 0.36) and a CRI of 87.^11^ Similarly, Sun *et al.* produced a white LED using CD and polymer dots with a CCT of 5821 K and CIE of (0.33, 0.34).^12^ These results suggested that increasing the proportion of warm light might be done either by growing CQDs with longer emission wavelengths by UV stimulation or by merging some efficient QDs with CQDs. It is difficult to make white LEDs with CQDs in combination with polymer dots or hybrid phosphors as colour converters.^13^

Fluorescence nanoparticles of a novel class of synthetic fluorescent materials with a particle size range of 1 to 10 nm is called carbon quantum dots (CQDs). They have excellent, high chemical stability,^14^ low toxicity,^15^ multi-colour tunable fluorescence^16^ and easy to functionalize. The most notable property of these nanoparticles is their luminous nature. As a result, CQDs have been extensively utilised in the domains of photocatalysis,^17^ solar technology,^18^ ion detection,^19^ bioimaging,^20^ and light-emitting devices.^21^ There are many ways to make CQDs; some of the more well-known routes include electrochemical oxidation,^22^ microwave irradiation,^23^ laser ablation,^24^ hot injection,^25^ hydrothermal,^26^ and pyrolysis.^27^ In this study, we used a one-step hydrothermal technique to synthesis CQDs from *Plumeria* acuminate plant leaves in alcoholic solvent (1-propanol). With excitation wavelength 370 nm *Plumeria* acuminate CQDs has prominent emission peak at 676 nm with a narrow band, and another relatively low intensity broad band emission from 350 nm to 480 nm.

WLEDs are essentially mixtures of different colours like RGB (red, green, blue). In this work, Tb^3+^ has been used as dopant in *Plumeria* acuminate CQDs to acquire the green emission spectra to fabricate WLEDs. Under UV excitation, Tb^3+^ produces intense green emission at 540 nm.^28^ The concentration of Tb^3+^ in CQDs has been optimized using photoluminescence (PL) emission spectra and the International Commission of Elcairage (CIE). The process, which involves fusing the acquired CQD + Tb^3+^ ions film with a UV chip (370 nm) to create white LEDs, is simple and inexpensive. This study produced functionalized CQDs with high QY and good RGB spectral composition, which were subsequently instantly converted into CQD + Tb^3+^ films. The white LED was then made using a single CQD + Tb^3+^ film and a UV chip.

Rare earth and transition metal ions have been used in numerous WLED investigations. However, the cost has not been found to be economical. As a result, attempts to produce WLEDs from natural resources are also being made, although various problems such as low quantum yield, brief emission lifetime, and self-quenching arise. We improved the properties of the WLEDs by combining carbon quantum dots (CQDs) from natural sources with rare earth ions to address these problems.

## 2. Experimental details

### 2.1. Materials

We use the following starting materials to synthesized samples, terbium acetate hydrate (99.99%), di-ionised water, 1-propanol, and fresh *Plumeria* plant leaves. All materials were used further without purification. These materials are purchased from Sigma Aldrich and *Plumeria* plant leaves taken from IIT(BHU) campus.

### 2.2. Methodology

Using the hydrothermal approach, we must prepare carbon quantum dots (CQDs) from leaves of the *Plumeria* plant. First, 38 grammes of neat, clean *Plumeria* plant leaves were taken and cut into small pieces. They were then added to 100 millilitres of 1-propanol and heated at 100 °C for three hours before being stored and safely kept in a hydrothermal autoclave. This autoclave was then placed inside a muffle furnace for 8 hours at 160 °C, cooled naturally at room temperature, and then the resulting solution was centrifuged and filtered through a 0.22 mm. This solution stored at 4 °C for the further application.

Second, make a 10 millimolar solution of the readily soluble Terbium acetate in DI water for use in the processing of samples. At various volume ratios, terbium acetate and CQDs were combined.

### 2.3. Approaches for characterization

Different spectroscopic methods were used to characterise the synthesised CQDs either with or without Tb^3+^. Using an FEI Tecnai G2 High-resolution Transmission Electron Microscope (HRTEM) equipped with a liquid nitrogen-cooled sample container, an image was obtained and its dimensions and shape were examined. The X-ray diffraction (XRD) spectra of CQDs were obtained using the benchtop X-ray diffractometer Rigaku Miniflex 600 Desktop X-ray Diffraction System, made by RIGAKU Corporation. In order to measure the Fourier-transform infrared (FTIR) spectra in KBr medium, a Jasco FR/IR-4600 spectrometer was employed. Thermo-scientific K-Alpha spectrometer was used to perform X-ray photoelectron spectroscopy (XPS) and evaluate chemical compositions. X-ray photoelectron spectroscopy was captured using the Axis Ultra system and a monochromatic Al K X-ray source (1486.6 eV). The energy resolution was set at 0.1 eV. The UV-vis absorption spectra were captured using an Eppendorf kinetic Bio-Spectrophotometer. An Eppendorf kinetic Bio-Spectrophotometer was used to record the UV-visible (UV-Vis) absorption spectra. The excitation and emission spectra of CQDs were recorded using a Fluorolog FL3C-21 of a Horiba spectrofluorometer with a Xe lamp as the excitation source. Time-resolved fluorescence decay curves were produced using a Horiba Jobin Yvon single-photon counting system with a 375 nm diode laser at a 1 MHz repetition rate and 1.3 ns pulse width. Using Origin2019b, we analysed the CIE coordinate and CCT of the emitting white LED, and we conducted EL experiments with QEpro (Ocean optics). The quantum yield (QY) of functionalized CQDs was achieved by using a comparative approach in accordance with a predetermined protocol.^52^ Relative quantum yield (QY) of the CQDs was also evaluated by comparing with a reference fluorophore, Rhodamine 6G, to determine the QY of the functionalized CQD solution (quantum yield of Rhodamine 6G is 95%). Each absorbance measurement for the solutions was taken at an excitation wavelength of 370 nm. The region under the photoluminescence curve for the wavelength range of 390–720 nm is referred to as the integrated emission intensity. Utilizing integrated emission intensity *vs.* absorbance, the plots were made. The QY of functionalized CQDs was determined using eqn (1).

1 *Q* = *Q*_st_(*K*/*K*_st_)(*η*/*η*_st_)

the subscript “st” stands for standard rhodamine 6G, and ‘*Q*’ is defined as quantum yield (QY), while ‘*K*’ is representing the slope of the integrated emission intensity with respect to absorbance plot of CQDs, *η*/*η*_st_ = 1.

## 3. Results and discussion

### 3.1. Optical properties characterization

**Fig. 1:**
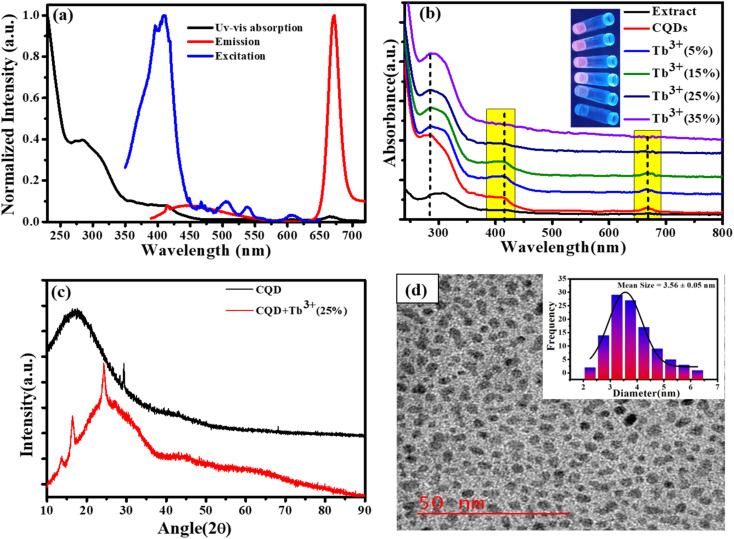
(a) UV-Vis absorption, excitation, and emission spectra of CQDs. (b) UV-vis absorbance of CQDs with various Tb^3+^ ion molar ratios (inset: the modification of CQDs emission colour under UV light). (c) XRD pattern of CQDs and CQDs + Tb^3+^(25%). (d) TEM image of CQDs inset: bar graph showing size distribution of CQDs.

The early optical characteristics of CQDs, including UV-Vis, excitation, and photoluminescence measurements, are shown in Fig. 1a. Two distinct peaks in the UV-Vis absorption may be seen: a very modest absorption band at 676 nm and a prominent absorption band at 280 and 410 nm. The π–π* transition of sp^2^ hybridised C=C bonds appears at 280 nm (ref. 29 and 30) while the 410 nm absorption is attributed to the C=O n to π* transition, which is caused by the π-plasmon and signals the beginning of the precursor's carbonization into nanostructures that resemble graphitic materials.^31^ The magnesium porphyrin derivative chlorophyll has a large π-electron system, and the electronic transitions between the π and π* orbitals of the chlorophyll macrocycle result in broad absorption bands between 380 and 430 nm (B band) and 600 to 800 nm (Q band). This results in the absorption peaks at 410 nm and 677 nm (Soret or B band).^32,33^ The fluorescence excitation spectrum at the specified emission wavelength describes the electron distribution of the molecule in its ground state and a broad range of electron distribution (350–450 nm) that matches the 676 nm emission wavelength was obtained. Here, we are selecting an appropriate excitation wavelength (370 nm) because of the CQDs shows the excitation dependent behaviour of emission spectra. These excitation wavelengths resulted in strong, intense emission at 676 nm, which was also obtained broad emission in the area of blue between 400 to 550 nm. The absorption spectra of CQDs containing Tb^3+^ ions at various molar ratios are presented in Fig. 1b. The inclusion of Tb^3+^ ions in CQDs at lower molar ratios (5%, 10%, and 15%) did not result in noticeably larger changes, but at higher molar ratios (20%,25%, and 35%), it was clearly visible that the Q band and 410 nm peak intensity decreased without affecting the peak location. This result indicates that complexes can form at higher Tb^3+^ ion molar ratios. Additionally, UV light application caused colour changes in CQDs containing different molar ratio of Tb^3+^ ions; this is shown in inset Fig. 1b. The XRD pattern of CQDs and CQDs containing Tb^3+^ ions are shown in Fig. 1c. The XRD patterns of CQDs feature a broad diffraction peak at 19° that represents their amorphous nature and a sharp peak at 28.42° that corresponds to the sp^2^ hybridised graphite carbon (002) plane.^34^ Tb^3+^ ion (25%) is also added to create a complex that improves crystallinity with a new crystal structure. (011), (210) and (022) plans in these freshly formed crystal structures correspond to 13.38°, 16.48°, and 24.44°, respectively.^35^ According to high resolution transmission electron microscopy (HR-TEM), the CQDs dimensions are as illustrated in Fig. 1d. The HR-TEM images show the homogeneous distribution of CQDs particle with quasi-spherical shape within ranging 2.5 to 5.5 nm and the bar graph in (Fig. 1d inset) shows that the average size of the CQDs is 3.56 nm.

### 3.2. FTIR and XPS measurements

**Fig. 2:**
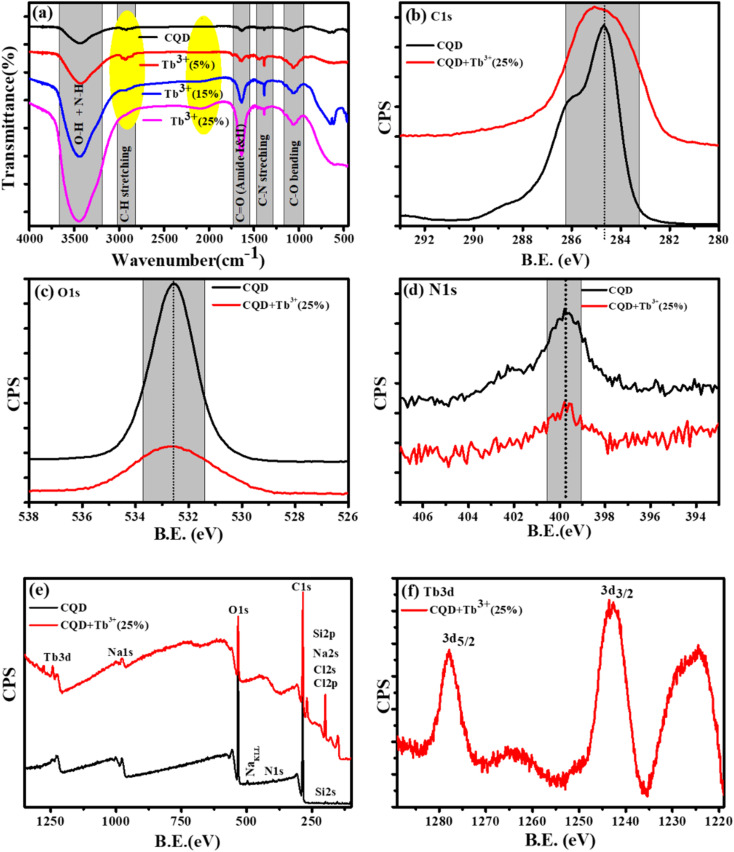
Spectroscopic characterisation of CQDs and CQDs + Tb^3+^(25%). (a) FTIR spectra with and without Tb^3+^ ions. High resolution XPS spectra of the photoelectron B.E. peaks at (b) C1s, (c) O1s, and (d) N1s. (e) XPS survey of CQDs and CQDs + Tb^3+^(25%). (f) Tb3d spectrum.

The X-ray photoelectron (XPS) and Fourier Transform Infrared Spectroscopy (FTIR) studies were made to better understand of the chemical composition and surface functionality of CQDs. The formation of the FTIR spectra of synthesised CQDs at 160 °C is depicted in Fig. 2a. We see wide and instance band absorption in the FTIR spectra between 3200 and 3800 cm^−1^, which are a sign of combined O–H and N–H stretching vibrations.^36^ The weak band identified at 2918 cm^−1^ is related to C–H stretching, while the broad, weak bands identified at 1642 cm^−1^ and 1380 cm^−1^ correspond to carbonyl groups C=O and C–N stretching, respectively.^37^ C–O vibrations can also be recognised by weak absorption at 1049 cm^−1^. This information demonstrates that the synthesized CQDs are adorned with several functional groups, many of which are rich in oxygen, carbon, and nitrogen.

The XPS spectra of the C1s, O1s, and N1s, corresponding the photo-electron peaks of the CQDs and CQDs + Tb^3+^(25%) are shown in Fig. 2b–d. Here, we found that for Tb^3+^(25%), the peak shape and peak position of C1s and O1s remained almost unchanged, indicating that there may be weak interaction with carbon and oxygen. With the addition of Tb^3+^(25%), the N1s XPS spectra show the same peak position. The XPS survey of CQDs and CQDs + Tb^3+^(25%) is presented in Fig. 2e. The survey spectrum reveals that the C and O species make up the majority of the CQDs, with the proportion of N species being the least. While C, O, N and Tb3d are present in CQDs + Tb^3+^(25%). The peaks at 1242.65 and 1277 eV in Fig. 2f are attributed to 3d_5/2_ and 3d_3/2_ of Tb^3+^ respectively, whereas the binding energy at 1224.9, 1264, and 1279.35 eV, which are related to Tb^4+^ (3d_5/2_), Tb^4+^ (3d_5/2_) and Tb^4+^ (3d3/2).^38^ According to the research, Tb is present in the CQDs + Tb^3+^(25%) in both Tb^3+^ and Tb^4+^ oxidation states.

**Fig. 3:**
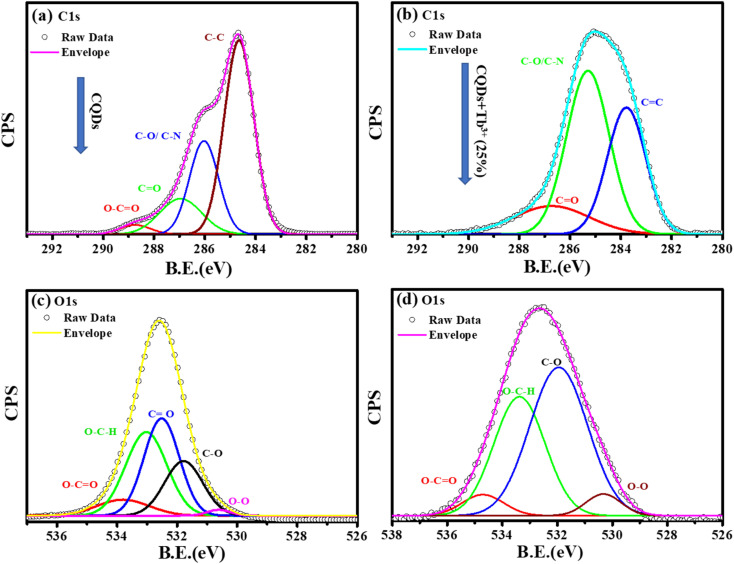
Deconvolution of C1s, and O1s photoelectron spectra of CQDs in graph (a), and (c) while CQDs + Tb^3+^(25%) is in (b), and (d).

To learn more about each of the several species that were found in the C1s, O1s, and N1s of the CQDs and CQDs + Tb^3+^(25%) fitted into numerous components. The peaks at 284.6, 286.04, 286.9, and 288.8 eV that are visible in the deconvoluted image of the C1s peak of CQDs and CQDs + Tb^3+^(25%) are assigned to the sp^2^ carbon atoms C–N/C–O, C=O, C–C/C=C, and O–C=O each, shown in Fig. 3a and b. We have referred to the C–O and C–N peaks as C–O/C–N since they could not be resolved. Two peaks in the N1s spectra are present at 399.6 and 402.2 eV and are associated with C–N and O=C–N, respectively. After adding Tb^3+^(25%), the surface passivation of CQDs involves the C–N and O=C–NH groups. Multiple oxygen species are present when the O1s peak is wider. According to the peak fitting of O1s Fig. 3c and d, the O1s peak can be divided into species with binding energies of 533.9, 533.03, 532.5, 531.7, and 530.5 eV respectively, of C–OH, O–C–O, C–O, and O–O.^39,40^

According to the FT-IR and XPS characterizations of the CQDs as they were obtained, Tb^3+^ is involved in the surface passivation of CQDs. Based on fluorescence, research was done to better comprehend the molar ratio of CQDs to Tb^3+^. We observed that the Tb^3+^(25%) molar ratio exhibits excellent fluorescence performance.

### 3.3. PL, QY and LT measurements

**Fig. 4:**
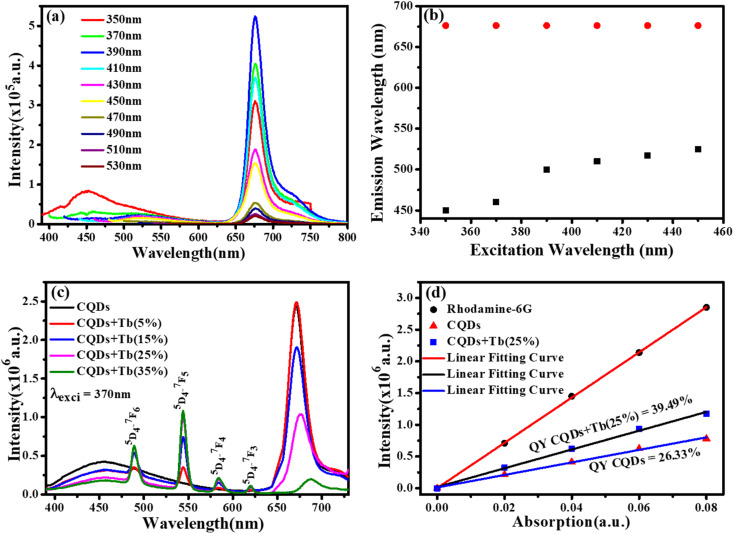
(a) Excitation dependent study of CQDs in 1-propanol solution. (b) Shows the relation between excitation (350–450 nm) and emission wavelength. (c) PL emission spectra of CQDs in 1-propanol solution with different molar ratio of Tb^3+^ ions. (d) QY diagram of CQDs and 25% Tb^3+^ doped CQDs.

We chose CQDs + Tb^3+^(25%) as the ideal molar ratio for use in the subsequent application based on PL intensity. Excitation-dependent analysis of CQDs + Tb^3+^(25%) was displayed in Fig. 4a, and it showed that the emission occurred in the blue area (400–550 nm) and red region (650–750 nm). Here, the blue region depicts the shift in emission caused by excitation, whereas the red region's peak position is observed to remain constant while the intensity changes shown in Fig. 4b, potentially as a result of CQDs uniform size and the distribution of identical or comparable on each passivated carbon dot are emissive sites.^41,42^ Unlike in past research when the PL peak changes to longer wavelengths as the excitation wavelengths are lifted.^43,44^ In consequence, the distribution of different-sized particles and emissive sites on CQDs^22,45^ might be reflected in their PL spectra. To ascertain which component has a substantial impact on the excitation-independent behaviour of the uniform morphology or the emissive spots on the CQDs. Additionally, a PL spectrum test of the CQDs using different molar ratios of CQDs and Tb^3+^ was conducted using the same excitation wavelength (370 nm), as depicted in Fig. 4c.

The emission associated with the electronic transition of Tb^3+^ ions (^5^D_3_ to ^7^F_*j*_ (*j* = 5, 4, 3)) increases as the molar concentration of Tb^3+^ ions rise, but at the same time, we see a decrease in the PL intensity of CQDs. This PL emission's adaptability to colour ratio adjustments displays the best colour ratio (red, green, and blue) for WLEDs, which corresponds to Tb^3+^(25%) doping. It also reveals that the CIE value is becoming closer to the standard value.

Quantum yield (QY) was estimated using Rhodamine 6G as a reference (QY = 95%). Fig. 4d depicts the graph between the prepared rhodamine6G, CQDs, and CQDs + Tb^3+^(25%) sample absorption and PL emission was plotted at the fixed excitation wavelength (370 nm) and the absorption does not exceed more than 0.1. To determine the slop in the quantum yield measurements of the CQDs, and CQDs + Tb^3+^(25%) with respect to rhodamine 6G and hit the QY 26.33% and 39.49%, were measured by eqn (2). The explanation for the high emission intensity and QY of the CQDs passivated by Tb^3+^ may be due to the reduction of the carboxylic groups, which serve as nonradiative recombination centres of electron–holes.^46^

**Fig. 5:**
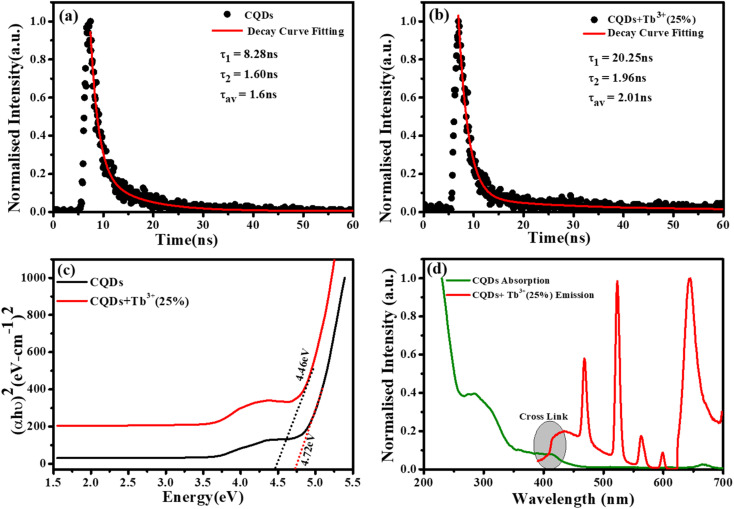
The lifetime–decay curves of (a) *Plumeria* plant leaves, (b) CQDs + Tb^3+^(25%) (the laser excitation source is 375 nm). (c) Optical band gap of with and without doped Tb^3+^(25%) ions. (d) The absorption spectra of undoped and emission spectra of dopped Tb^3+^(25%) ions.

To measure the fluorescence lifetime behaviour of CQDs and further look into how Tb^3+^ affects the photoluminescence of CQDs, the multidimensional time-correlated single photon counting (TCSPC) approach was used. As a result, the fluorescence lifetime of CQDs made from the leaves of the *Plumeria* plant was used as a benchmark. The two decay curves were modelled by biexponential functions *R*(*t*) in eqn (2), as shown in Fig. 5a and b:

2 *R*(*t*) = *A*_1_ e^(−*t*/*τ*_1_)^ + *A*_2_ e^(−*t*/*τ*_2_)^

where *A*_1_, *A*_2_ are the time-resolved decay lifespan pre-exponential factors, *τ*_1_ and *τ*_2_ represent the resolve decay lifetime. The average lifetime is then calculated using eqn (3):3
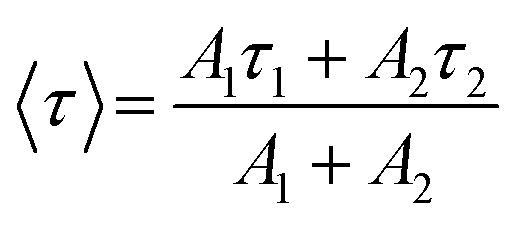


The average lifetime was calculated of the entire fluorescence decay process of the target CQDs is 1.6 ± 0.02 ns (Fig. 5a), which is less than CQDs + Tb^3+^(25%) (2.01 ± 0.05 ns) (Fig. 5b). These lifetimes were calculated corresponding to 676 nm emission peak and used 370 nm of the diode LASER as an excitation source. The fractions of *τ*_1_ and *τ*_2_ are 83.8% and 16.19% for the CQDs produced from the leaves of the *Plumeria* plant, while they are 91.17% and 8.82% for the target CQDs. When the Tb^3+^ ions are linked to the CQDs, the fraction with the longer lifetime (*τ*_1_) increases. The longer fluorescence lifetime demonstrates both the fluorescence quantum yield of CQDs as well as how Tb^3+^ions contribute to the fluorescent stability. The outcomes might be explained by a decrease in nonradiative traps^47,48^ or an increase in emissive sites^49^ resulting from the passivation of the surface by Tb^3+^ ions. Additionally, the “rapid” decay (*τ*_2_) may be triggered by the exciton transition between the surface groups, while the “slow” decay (*τ*_1_) may result from the exciton transition from the carbogenic core to the surface groups.^50^

By extrapolating from the eqn (4), it is found in Fig. 5c that the apparent optical band gap of Tb^3+^(25%) that is undoped and doped is approximately 4.72 and 4.46 eV.

4 (*αhν*) = *C*(*hν* − *E*_g_)^*n*^

where *C* is a constant, *E*_g_ is the material's average band gap, *α* is the molar extinction coefficient, and *n* is dependent on the kind of transition. *E*_g_ in eqn (3) stands for direct allowable band gap for *n* = 1/2.

**Fig. 6:**
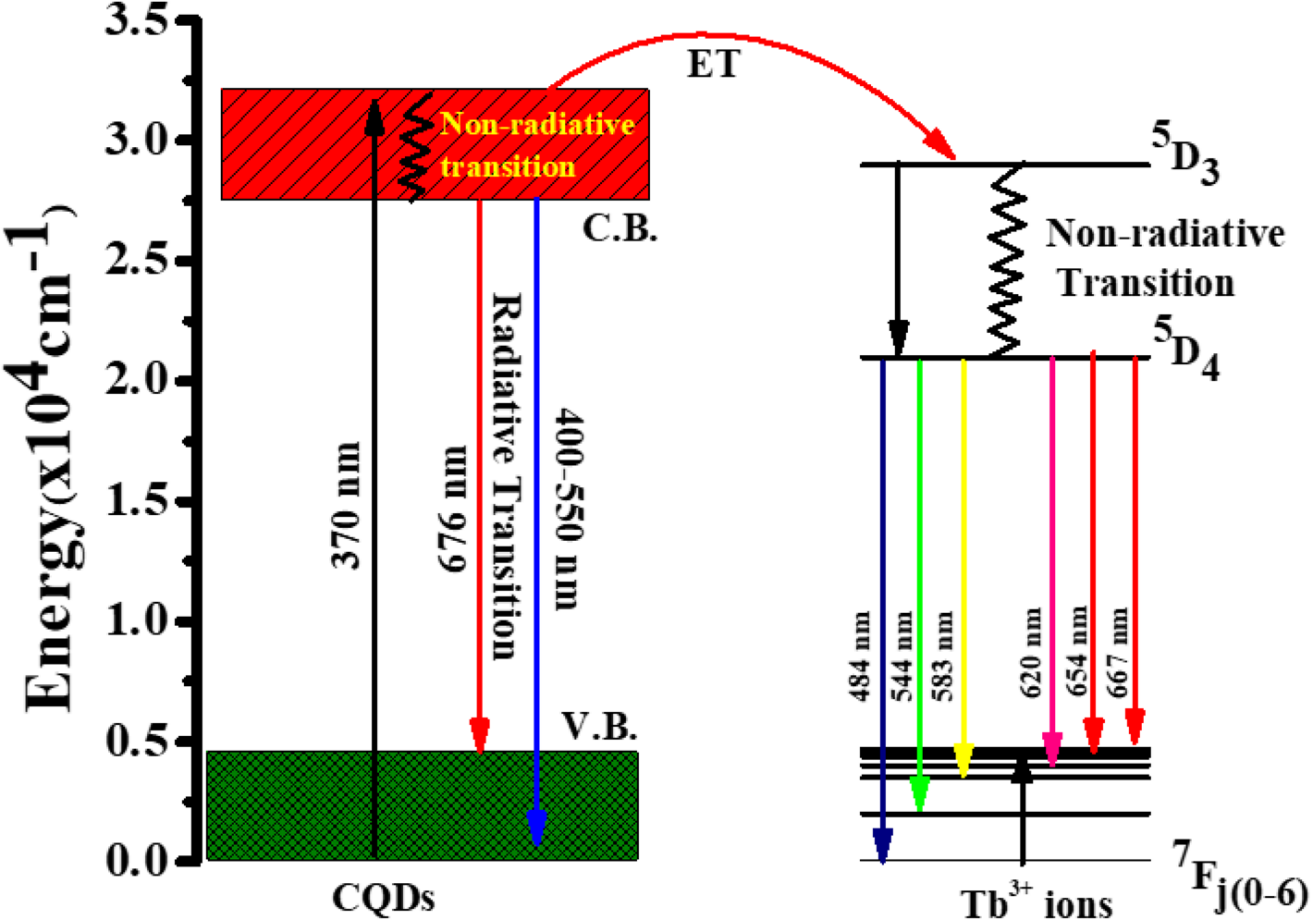
Energy transfer mechanisms from the host CQD to the 4f shell of Tb^3+^ ions, included cross relaxation between ^5^D_3_ → ^5^D_4_ and ^7^F_6_ → ^7^F_0,1_, phonon-assisted non-radiative transition of ^5^D_3_ → ^5^D_4_, and radiative transition of ^5^D_4_ → ^7^D_*j*_ (*j* = 6, 5, 4, 3).

Fig. 5d represent the absorption spectra of doped and emission spectra of undoped Tb^3+^(25%) ions, the donor and acceptor molecules are separated by a certain distance, which is normally 1 to 10 nm,^51–53^ but this example shows donor dipole–acceptor dipole overlapping of the molecules. This overlapping has been confirmed fluorescence resonance energy transfer (FRET) mechanism. Tb_2_O_3_ has an absorption band between 200 and 400 nm, with its primary absorption peak near 225 nm attributed to Tb^3+^ ion 4f8 → 4f^7^ electronic transitions.^54^ However, compared to undoped CQDs, this absorption at the excitation energy is substantially weaker. So, while the 554 nm emission is primarily driven by the photon absorption of the CQDs host and the energy transfer process from host material to rare earth ions is thought to exist in the doped CQDs, we assume that the absorption of terbium acetate hydrate may slightly contribute to the excitation of Tb^3+^ ions.^55^ In this study, we provide a mechanism for energy transfer from the host CQD to the Tb^3+^ ions. According to Fig. 6, photon absorption causes the electrons in CQDs to move from the valence to the conduction band. This energy is subsequently transmitted non-radiatively to the ^5^D_3_ level of Tb^3+^ ions. The excited electrons in the ^5^D_3_ level can passage to lower levels in three different ways: one is the radiative transition from ^5^D_3_ → ^7^F_*j*_ (*j* = 5, 4, 3), which corresponds to three emission lines of 410, 432, and 467 nm that merge in the CQDs PL emission in the range of 390–500 nm; the other is the cross relaxation between ^5^D_3_ → ^5^D_4_ and ^7^F_6_ → ^7^F_1,0_ due to the similar energy gap. The transition of ^7^F_6_ → ^7^F_1,0_ in a nearby Tb^3+^ ion absorbs the energy generated by the cross-relaxation mechanism, which converts excited ^5^D_3_ states into ^5^D_4_ states.^55–57^ The final transition, which is crucial to the system, is the nonradiative transition of ^5^D_3_ → ^5^D_4_, which is multi-phonon assisted. As a result, the chance of the ^5^D_3_ state emitting radiation is minimal, and the majority of the excited electrons in the ^5^D_3_ level are depopulated into the ^5^D_4_ level. The emission lines of 493, 554, 590, and 624 nm, respectively, correspond to the radiative transitions from ^5^D_4_ → ^7^F_*j*_ (*j* = 6, 5, 4, 3) transition.

### 3.4. Creation of white LEDs

The UV chip with peak emission centred at 370 nm was employed as a subtractor, fixed at the bottom of the base, and connected to an external power source because we are interested in creating WLEDs. Then, after optimising the inner surface coating of the optical lens with various volume concentrations of 25% Tb^3+^ in CQDs, the lens is placed in the oven at 80 °C for three hours. Finally, our obtained optical lenses were firmly mounted to the LED chip's underside to enable the creation of a cold white LED.

### 3.5. WLEDs demonstration

**Fig. 7:**
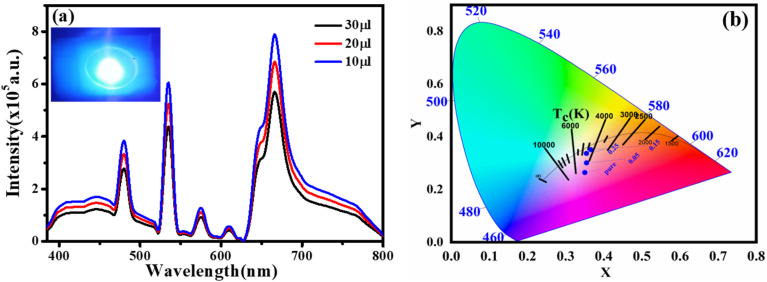
(a) EL spectrum of the WLEDs with distinct thickness of the CQDs + Tb^3+^(25%). Inset: WLEDs demonstration. (b) CIE chromaticity plot for the WLEDs at various molar concentration of Tb^3+^ ions (0%, 5%, 15%, and 25%).

We created a white LED using the obtained CQD + Tb^3+^(25%) film used as a single generator for white light, effectively converting UV light into white light, taking into account the excellent qualities the synthesised functionalized CQDs exhibit, such as strong PL intensity, high QY value, and good film-forming ability.^58^ This white LED has a fairly broad emission spectrum that covers practically the whole visible spectrum (400 to 800 nm) (Fig. 7a), which has a wider emission of CQDs under UV stimulation. The behaviour differs from earlier results where a strong UV light from the chip was present.^44,59^ After absorbing greater energies, fluorescence often manifests itself at lower energies or longer wavelengths. Additionally, fluorophores may cause additional Stokes shifts as a result of environmental factors such complex formation, solvent effects, or energy transfer.^52^ When compared to the PL spectra of CQD solution, the LED using CQDs as the phosphor exhibits two distinct peaks at 448 and 580 nm. This effect may be explained by the solid-state CQDs integrated in the LED lens transferring energy through light reabsorption.^60,61^ Some CQDs are excited by the UV chip to produce light with a shorter wavelength (448 nm). The light is then partially reabsorbed by the CQDs in the area, and longer wavelength light is released from those CQDs. This offers an adequate justification for the peak centred at 580 nm.

The constructed WLED exhibits the ability to meet the luminous requirement and be used in both indoor and outdoor lighting systems by producing brilliant, cool white light at a current of 20 mA and 3.7 V.^62^ According to Fig. 7b, the CIE chromaticity coordinates of (0.35,0.26), (0.35,0.30), (0.35,0.35), (0.33, 0.34), and (0.38, 0.41) correspond to CQDs, 5% Tb^3+^ doped CQDs, 15% Tb^3+^ doped CQDs, 25% Tb^3+^ doped CQDs, and 35% Tb^3+^ doped CQDs, respectively. Here, 25% Tb^3+^ doped CQDs have CIE closest to-standard value of (0.33, 0.34). Further, the colour correlated temperature (CCT) of 25% Tb^3+^ doped CQDs is 4995 K with CRI 84.2%, which is consistent with cool white. According to these specifications, CQDs offer a lot of promise for use in solid-state lighting systems.

The injection volume of CQD solution determines the thickness of the CQD layer on the LED's optical lens. Fig. 7a shows how the CQD layer thickness affects the device's emission spectra when operating at 3.5 V. When the small amount of CQD solution also drops, the emission intensity does as well. The drop in LED emission intensity is caused by either an increase of nonradiative traps or a decrease in LED light transmittance, both of which cause more thermal radiation to be produced throughout the energy transfer process *via* light reabsorption under specific illumination levels.

## 4. Concusions

In conclusion, we have synthesized fluorescent functionalized carbon quantum dots (CQDs) by a facile one step hydrothermal method at 160 °C for 8 h using *Plumeria* plant leaves as precursor and Tb^3+^ ions as a surface passivator. The prepared doped CQDs exhibit excitation-dependent PL emission and obtained strong peaks at 493, 554, 590, and 624 nm, respectively, is due to doping of Tb^3+^ ions. The CQDs and CQDs + Tb^3+^ ions have amorphous in nature with narrow size distribution and show high fluorescence quantum yield (26.33%, and 39.49%). The additive agent Tb^3+^ has engaged in reaction following the synthesis of the CQDs, as shown by the structural characterisation of the CQDs studies. We paired the single CQD phosphor film with the UV chip to create a WLED that emits cool white light with a CIE coordinate of (0.33, 0.34), a corresponding colour temperature of 4995 K with CRI 84.2, and favourable electroluminescence behaviour as a white-light converter for white LED.

## Conflicts of interest

Authors have no conflicts to declare.

## Supplementary Material
